# *Acanthamoeba* spp. in Urine of Critically Ill Patients

**DOI:** 10.3201/eid1507.081415

**Published:** 2009-07

**Authors:** Leonilda C. Santos, Maura S. Oliveira, Renata D. Lobo, Hermes R. Higashino, Silvia F. Costa, Inneke M. van der Heijden, Mauro C. Giudice, Atalanta R. Silva, Anna S. Levin

**Affiliations:** Itaipu Binacional and Universidade do Oeste do Paraná, Paraná, Brazil (L.C. Santos); University of São Paulo, São Paulo, Brazil (M.S. Oliveira, R.D. Lobo, H.R. Higashino, S.F. Costa, I.M. van der Heijden, M.C. Giudice, A.R. Silva, A.S. Levin)

**Keywords:** Acanthamoeba, parasites, urinary tract infections, nosocomial infections, cross-sectional studies, intensive care units, letter

**To the Editor:** Free-living amebae are ubiquitous protists able to endure extreme temperature and pH in diverse environments (*1*). In past decades, interest in them increased as causes of infections, such as keratitis (*2*) and granulomatous encephalitis (*3*). *Acanthamoeba* spp. can harbor pathogenic microorganisms as endosymbionts, such as bacteria (e.g., *Legionella* spp., *Pseudomonas aeruginosa*, and *Vibrio cholera*), fungi, and mycobacteria (*4*).

The occasional observation of amebae in urine specimens in a clinical microbiology laboratory (by L.C.S.) motivated our study. To estimate the prevalence of *Acanthamoeba* spp. in urine, on March 3 and 4, 2008, we collected urine samples from indwelling urinary catheters of all critically ill patients in the intensive care units of a tertiary care 2,000-bed university hospital (Hospital das Clínicas, University of São Paulo). Medical records were evaluated for patient age, sex, underlying diseases, length of hospital stay, use of central venous catheter, mechanical ventilation, antimicrobial drug use, and duration of urinary catheterization.

Chemical urinalysis was performed by using a urine dipstick (Uriquest; Labtest Diagnostica, Lagoa Santa, Minas Gerais, Brazil), and urine leukocyte and erythrocyte counts were performed. Samples were examined microscopically and cultured for amebae. Amebae were characterized on the basis of morphologic criteria (cyst morphology and trophozoite shape and motility) (*5*). For ameba culture, 10 mL of urine was centrifuged at 2,500 rpm for 5 min. The supernatant was discarded, and the sediment (1 mL) was added to 5 mL of brain-heart infusion (Oxoid, Cambridge, UK). Cultures were incubated at 25^o^C for 48 hr and microscopically examined. For this study, finding trophozoites on direct examination or growing *Acanthamoeba* on culture were considered a positive result for the patient.

Pipet tips (10), vacuum containers (10), plastic 15-mL tubes (10), syringes (10), glass slides (20), and tubes containing medium (10) were submitted for direct examination and culture for ameba to ensure that they were not contaminated. Urine samples were cultured for bacteria and fungi. Urine samples were submitted for bacterial and fungal direct examinations, and cultures were performed (*6*). If found, organisms were identified by morphologic, biochemical, and Gram stain characteristics.

Data from patients with and without *Acanthamoeba* spp. were compared. For dichotomous variables, we used χ^2^ to calculate odds ratios and 95% confidence intervals. For continuous variables, the Mann-Whitney test was used. Results were significant at p<0.05.

A total of 63 urine samples were evaluated; 17 (26%) were positive for *Acanthamoeba* spp. (Table). All samples of the control materials and medium tested showed negative results.

The high prevalence of *Acanthamoeba* spp. in the urine of critically ill patients is difficult to explain. Although *Acanthamoeba* spp. can cause severe infections, amebae also carry pathogenic microorganisms (*4*). Bacteria may serve as food for amebae, but other interactions exist; for example, bacteria take advantage of the protection offered by amebae, especially in the cystic form (*4*). *P. aeruginosa*, *Escherichia coli*, and *Proteus mirabilis* can infect free-living amebae (*7*). The presence of *Acanthamoeba* spp. in critically ill patients may be advantageous to potentially pathogenic bacteria in the urine, protecting them against antimicrobial drugs, disinfectants, and host immunity. In this sense *Acanthamoeba* spp. may be a reservoir for pathogenic bacterial agents in severely ill patients or, as Khan described, a “Trojan horse for bacteria” (*1*). However, we found no association between the presence of *Acanthamoeba* spp. of bacteria and fungi in the urine.

Biofilms are attractive niches for *Acanthamoeba* spp. that may provide nutritional requirements and protection against disinfectants and antimicrobial drugs (*1*). After 7 days in a patient, most urinary catheters contain a biofilm (*8*). However, we found no association between duration of catheterization and presence of *Acanthamoeba* spp. Also, *P. aeruginosa* isolates from clinical infections have shown more virulence toward *Acanthamoeba* spp. than environmental samples (*9*).

Another possible explanation is the direct pathogenic activity of *Acanthamoeba* spp. In a study to determine whether ameba-associated microorganisms are a cause of nosocomial pneumonia, in 5 of 210 cases, *Acanthamoeba* sp. was considered the only cause of infection (*10*). In our study, patients positive for *Acanthamoeba* spp. had a higher mean and median of urine leukocytes and erythrocytes, suggesting aggression by the amebae. On the other hand, even these higher counts among positive patients are considered relatively low. Cardiovascular disease, cancer, and diabetes were associated with carriage of *Acanthamoeba* spp., which may occur in more severely ill patients. As a final possibility, *Acanthamoeba* spp. in the urine could have no role at all and may even reflect contamination during catheterization.

This study has limitations. It was a small, preliminary investigation designed only to evaluate the presence of *Acanthamoeba* spp. in urine. However, our findings should lead to further studies to increase knowledge about the role of free-living amebae in nosocomial infections.

**Table:** Univariate analysis of variables potentially associated with *Acanthamoeba* spp. in urine samples from critically ill patients, Hospital das Clínicas, University of São Paulo, Brazil, March 2008*

Variable	Sample positive for *Acanthamoeba* spp.†	Sample negative for *Acanthamoeba* spp.‡	OR	95% CI	p value
No. (%) patients					
Male sex	10 (59)	28 (61)	0.92	0.30–2.95	0.88
Antimicrobial drug use	13 (77)	35 (76)	1.02	0.28–3.78	0.97
Use of mechanical ventilation	10 (59)	23 (70)	1.43	0.46–4.40	0.53
Presence of central venous catheter	12 (75)§	33 (72)	1.18	0.32–4.34	0.80
Urine culture positive for bacteria/fungi					
Any count	9 (53)	21 (46)	1.34	0.44–4.09	0.61
>10^5^ CFU/mL	8 (47)	13 (28)	2.26	0.72–7.12	0.16
Underlying diseases					
Cardiovascular	15 (88)	26 (57)	5.77	1.06–41.34	0.02
Infectious diseases	10 (59)	21 (46)	1.70	0.48–6.09	0.35
Cancer	9 (53)	9 (20)	4.63	1.20–18.40	0.01
Diabetes mellitus	5 (29)	3 (7)	5.97	1.02–38.03	0.02
Renal Insufficiency	2 (12)	6 (13)	0.89	0.11–5.81	0.89
Acute abdomen	2 (12)	4 (9)	1.40	0.16–10.44	0.71
Trauma	1 (6)	11 (24)	0.20	0.01–1.74	0.11
Respiratory	1 (6)	5 (11)	0.51	0.02–5.24	0.55
Neurologic	1 (6)	4 (9)	0.66	0.03–7.19	0.71
Others	8 (47)	19 (35)	1.26	0.36–4.45	0.68
Age, y					
Mean (SD)	62.4 (13.9)	54.7 (16.2)			0.05
Median (range)	64.6 (20.5–77.5)	53.4 (17.7–80.8)			
Length of hospital stay, d					
Mean (SD)	16.3 (12.8)	13.5 (11.3)			0.45
Median (range)	11 (2–43)	9 (1–48)			
Length of ICU stay, d					
Mean (SD)	6.9 (9.0)	9.4 (10.4)			0.18
Median (range)	3.0 (1–31)	5.5 (0–47)			
Duration of urinary catheterization, d					
Mean (SD)	8.3 (9.8)	10.4 (9.4)			0.17
Median (range)	4.0 (1–33)	8.5 (1–33)			
Leukocyte count in urine per high-power field					
Mean (SD)	10.9 (17.4)	3.59 (10.53)			0.009
Median (range)	3.5 (0–60)	1.0 (0–70)			
Erythrocyte count in urine per high-power field					
Mean (SD)	35.8 (42.6)	17.7 (27.9)			0.03
Median (range)	18.5 (0–150)	4.0 (0–120)			
Urine pH					
Mean (SD)	5.4 (0.8)	5.8 (1.1)			0.18
Median (range)	5 (5–8)	5 (5–8.5)			

*OR, odds ratio; CI, confidence interval. †n = 17 except as indicated. ‡n = 46. §n = 16.
